# Miscellany

**Published:** 1905-05

**Authors:** 


### Miscellany.

Dr. Endelman on Stomatitis.—A careful and intelligent consideration of the several factors involved in the complicated problem of nutrition with the consequent effect upon the body's defensive forces at once points out the etiological solution of nearly all inflammatory conditions of the oral mucous membrane. These inflammations of the lining membrane of the mouth I have divided into two classes,—those entirely localized to the areas of the oral cavity and those in which the lowering of the vital powers of the oral tissues brought about by some constitutional disorder has rendered it possible for the exciting factor (the bacteria then present in the mouth) to exercise its pathogenic influence.

The localized type of buccal inflammation I have named stomatitis simplex, and that in which a constitutional disorder acts as the predisposing cause, stomatitis complex.

Under the heading of stomatitis simplex I can include only those cases in which the inflammatory disorder is the immediate consequence of localized irritation, such as salivary deposits, carious cavities, rough edges of broken-down teeth, ill-fitting appliances intended for substitutes for the lost natural organs, also prolonged contact of the mucous membrane with substances of extreme temperatures, chemicals, tobacco smoke, etc. By the term stomatitis complex, I refer to all inflammations of the buccal mucous membrane in which the predisposing cause, an existing constitutional pathological error, has so modified the tissues of the mouth as to favor the growth and action of the microbic exciting cause. Here I include aphthous stomatitis, tuberculous stomatitis, ulcerative stomatitis, gangrenous stomatitis, mercurial stomatitis, syphilitic stomatitis, with stomatitis occurring in the course of the eruptive fevers. The so-called erythematous and catarrhal stomatitis may be considered as the primary stages of oral disturbances which if not eradicated at once may degenerate into either one of the above mentioned types of buccal and gingival inflammations.—*Dental Cosmos.*

---

Business and Profession.—A business, in its essence, consists in buying and selling—buying in the cheapest, and selling in the dearest, market.

A profession, in its essence, consists in accomplishing the end toward which special skill and special training is directed.

Take one or two simple examples. Merchants, shopkeepers, stock-brokers, are business men, whose main object it is to make as large a turnover as possible; that is to say, all their energies are primarily directed toward the making of profits and the acquisition of wealth. On the other hand, a student of science, an artist, or a physician, these are professional men, who, though they obtain a livelihood by means of their work, are compelled by the very nature of their work to put their work first, and the remuneration they receive for it second. The scientific man, if he is worthy of the name, must make his observations, his investigations, his experiments, his first consideration; the artist, he who is possessed of the creative faculty, if he has any real gift or inspiration, cannot help putting first his picture, his statue, his poem, his sonata; and so also the physician, whose work consists in the application of the healing art—he, unless he is prepared to forfeit the name of professional man, unless he is prepared to prostitute his calling by making it a mere shekel-grinding machine—he must ever direct his energies in the first instance to the healing of sickness, to grappling with disease and death. In other words, he must make his patients' well-being his first consideration. And he must do this, not from a quixotic notion that there is anything derogatory in accepting remuneration for services rendered, but he must do it simply because unless he puts his patients' interests first he will find that the quality of his work will suffer. If a physician makes his pocket, rather than his patient, his first consideration, it is as certain that his work will deteriorate as it is certain that the earth goes round the sun. For if he assumes the unprofessional attitude of the business man who looks first to the profit that is to be made, you will find inevitably that his judgment becomes warped, his knowledge becomes perverted, the edge of his skill becomes dulled and blunted, and he will constantly be taking an easier way than the right way for dealing with his cases, in order to save himself time and trouble and expense. Why is this? Simply because the interests of a man's pocket and the interests of his patients so frequently clash—they are not infrequently diametrically opposed; and so, unless he resolutely keeps before him his patients' welfare as his first, his prime concern, he cannot escape the temptation continually besetting the path of the practitioner to let "second best" stand in the place of "best," and to value a well-filled purse more than the satisfaction that is to be found in rendering faithful service.—L. MATHESON, L.D.S., *Dental Record*, England.

---

DR. RUDEL'S DETACHABLE WEDGE SEPARATOR.—The object of this apparatus is to facilitate placing the separating wedge in position. It is provided with eight steel wedges, any one of which may be forced between the teeth by screw pressure, after which the apparatus is removed, leaving the wedge in position. Each wedge is provided with a perforation through which a wire may be passed to prevent its slipping back after the pressure is released, and also with a shoulder over which the apparatus may be hooked to facilitate its removal. There seems to be no provision to prevent the wedge pressing unduly into the soft tissues.

Dental Examining Glass (designed by Dr. W. H. Williamson).—This is a lens of one-inch focus enclosed in a round metal tube to prevent as much as possible the breath clouding its surface; it may be stood on either end, and is fitted with studs to prevent its rolling when laid down. It overcomes some objections to the usual watchmaker's lens.

---

An Everted Rim Tumbler (designed by Dr. W. A. Hunt).—This is an ordinary tumbler with the edge or rim everted or turned outward; it is said that when so shaped the tumbler comes far more easily to the lips, and is more convenient for feeble patients, or those just recovering from an anæsthetic.

---

Tulloch's Alloy and Mercury Measures are intended as a ready means of measuring the amount of alloy and of mercury to be used in mixing for amalgam. The mechanism of the device is not described in the advertisement; as the price is high, it is probably complicated.

---

Fountain Spittoon.—C. J. Plucknett & Co., 28–37 Poland Street, London, W., offer a fountain spittoon to be attached to the wall by a swinging bracket. This has the advantage that all water and waste attachments are made at the wall line, and that it can be swung entirely clear of the chair. The writer used one somewhat similar for years with satisfaction. While the device advertised uses a flexible tube for the waste, the construction may be simplified by making the waste-pipe of the spittoon form the pivot of the bracket, connecting with the drain by a ground joint.

---

An Automatic Cylinder, for ethyl chloride, graduated on its side, and provided with a stop-cock operated by a gentle pressure with one finger, to secure accuracy of dosage and ease of administration.

